# Population-based correlates of COVID-19 infection: An analysis from the DFW COVID-19 prevalence study

**DOI:** 10.1371/journal.pone.0278335

**Published:** 2022-12-01

**Authors:** Amit G. Singal, Andrew Masica, Kate Esselink, Caitlin C. Murphy, Jill A. Dever, Annika Reczek, Matthew Bensen, Nicole Mack, Ellen Stutts, Jamie L. Ridenhour, Evan Galt, Jordan Brainerd, Noa Kopplin, Sruthi Yekkaluri, Chris Rubio, Shelby Anderson, Kathryn Jan, Natalie Whitworth, Jacqueline Wagner, Stephen Allen, Alagar R. Muthukumar, Jasmin Tiro

**Affiliations:** 1 University of Texas Southwestern Medical Center, Dallas, Texas, United States of America; 2 Texas Health Resources, Fort Worth, Texas, United States of America; 3 RTI International, Washington, District of Columbia, United States of America; 4 RTI International Headquarters, Research Triangle Park, North Carolina, United States of America; Augusta University, UNITED STATES

## Abstract

**Background:**

COVID-19 has resulted in over 1 million deaths in the U.S. as of June 2022, with continued surges after vaccine availability. Information on related attitudes and behaviors are needed to inform public health strategies. We aimed to estimate the prevalence of COVID-19, risk factors of infection, and related attitudes and behaviors in a racially, ethnically, and socioeconomically diverse urban population.

**Methods:**

The DFW COVID-19 Prevalence Study Protocol 1 was conducted from July 2020 to March 2021 on a randomly selected sample of adults aged 18–89 years, living in Dallas or Tarrant Counties, Texas. Participants were asked to complete a 15-minute questionnaire and COVID-19 PCR and antibody testing. COVID-19 prevalence estimates were calculated with survey-weighted data.

**Results:**

Of 2969 adults who completed the questionnaire (7.4% weighted response), 1772 (53.9% weighted) completed COVID-19 testing. Overall, 11.5% of adults had evidence of COVID-19 infection, with a higher prevalence among Hispanic and non-Hispanic Black persons, essential workers, those in low-income neighborhoods, and those with lower education attainment compared to their counterparts. We observed differences in attitudes and behaviors by race and ethnicity, with non-Hispanic White persons being less likely to believe in the importance of mask wearing, and racial and ethnic minorities more likely to attend social gatherings.

**Conclusion:**

Over 10% of an urban population was infected with COVID-19 early during the pandemic. Differences in attitudes and behaviors likely contribute to sociodemographic disparities in COVID-19 prevalence.

## Introduction

COVID-19, caused by severe acute respiratory syndrome coronavirus 2 (SARS-CoV-2), has resulted in over 6 million deaths worldwide, including over 1 million in the U.S., as of June 2022. The pandemic has tested public health infrastructures beginning in December 2019 with early reports from Wuhan, China [1, 2]. By mid-March 2020, COVID-19 had spread to all 50 U.S. states and the District of Columbia [3], with initial spikes in infections, hospitalizations, and deaths in large, diverse urban areas such as New York City [3, 4]. Although vaccines became available in December 2020, uptake has been slow in many areas for reasons including mistrust in the vaccine development process and belief that vaccines are unnecessary. Therefore, additional spikes in infections and deaths continue both globally and in the U.S.

Throughout the pandemic, even after introduction of the vaccines, there has been a clear need to understand prevalence and risk factors to inform public health interventions that can reduce transmission. Further, these data can help determine the number of persons who may be at risk for post-acute sequelae of COVID-19. Estimates of prevalence have varied widely (1–23%) due to sampling method (random selection [5–11], residual blood specimen testing [5, 12–14], convenience [15–20]), geographic coverage area (county [9, 10, 15–18, 20, 21], state [6, 8, 11, 19], national [5, 7, 12–14]), and timing of data collection (early [9–11, 13–17, 19–21] during the lockdown period vs. later [5–7, 12, 18]). Heterogeneity in the representativeness of these data and lack of information on correlates of infection make it difficult for local policymakers to plan public health efforts. This was particularly true for estimates of infection early in the pandemic, given limited and disparate access to testing, especially in racial/ethnic minority and low-income communities.

We launched a study in July 2020, involving a self-administered questionnaire and COVID-19 PCR and antibody testing, to estimate the prevalence of COVID-19, risk factors of infection, and related attitudes and behaviors (e.g., masking, physical distancing) in a racially, ethnically, and socioeconomically diverse urban population.

## Methods

The DFW COVID-19 Prevalence Study was led by the University of Texas Southwestern Medical Center and Texas Health Resources and conducted with RTI International. The Institutional Review Board of the University of Texas Southwestern Medical Center (STU-2020-0540) approved the study protocol that detailed informed consent obtained before the questionnaire and before COVID-19 testing.

### Study population

Protocol 1 of the DFW COVID-19 Prevalence Study was conducted from July 2020 to March 2021 on a randomly selected sample of adults aged 18–89 years residing in Dallas or Tarrant Counties, Texas. Dallas and Tarrant are the two most populous counties (2.6 and 2.1 million, respectively) in the Dallas-Fort Worth metropolitan statistical area. Across both counties, adults (age ≥18 years) comprise about 74% of the population, and the majority are racial and ethnic minorities. For example, as of July 1, 2019, the U.S. Census Bureau estimates that Blacks or African Americans comprise 24% of the population in Dallas and 18% in Tarrant County, and Hispanic/Latinx populations comprise 41% and 30%, respectively. Median household income in Dallas and Tarrant Counties was $59,607 and $67,700 in 2019, respectively, with 14% and 10% of residents living below the federal poverty line.

Eligible adults were randomly chosen through a two-stage sampling design. First, we randomly selected addresses from RTI’s Enhanced Address-Based Sampling (ABS) Frame (http://abs.rti.org/). The ABS frame comprises delivery points from the U.S. Postal Service’s Computerized Delivery Sequence file with monthly enhancements of ancillary data from both public (e.g., American Community Survey data from the U.S. Census Bureau) and private (e.g., predicted household composition) sources [22–24]. Second, we used county-specific Census block groups (CBGs) to classify addresses into eight mutually exclusive groups (i.e., strata) defined by socioeconomic status (SES; low, not low), Hispanic population density (low, not low), and non-Hispanic (NH) Black population density (low, not low). CBGs in each county with an average relative ranking of per capita income (Dallas: $16,113; Tarrant: $19,042) and percent of owner-occupied housing units (Dallas: 28.8%; Tarrant: 44.9%) below the 25^th^ percentile were classified as low SES. CBGs comprising less than 30% Hispanic residents were labeled as low-density Hispanic. Owing to differences by county, Dallas CBGs with less than 30% NH Black and Tarrant CBGs with less than 35% NH Black were designated as low-density NH Black. In each county-specific stratum, the two-stage sampling was designed to: (1) address estimated patterns of nonresponse, (2) minimize variation due to disproportionate sampling, and (3) achieve an initial goal of 13,500 participants per county, assuming a 40% overall response rate [25].

### Recruitment

Recruitment spanned July to October 2020. All randomly selected addresses were mailed a study invitation printed in English and Spanish that described the study and provided a toll-free number and website to request additional information or opt-out. The letter instructed a self-designated person at each address to identify one adult resident for the study using one of two methods randomly assigned to the address: 1) person with the most recent birthday; or 2) person meeting randomly assigned criteria combining age and sex (e.g., oldest female, youngest male). Reminder postcards were sent one week after the invitation letters to enhance participation. Trained interviewers also called phone numbers matched to each address, as available, around the time of the postcard mailing to promote study participation [26]. Nonresponding invitees in an initial subsample were also mailed a hardcopy questionnaire after the postcard but this did not increase response.

### Data collection

Study participants were asked to complete a 15-minute questionnaire. The questionnaire was programmed in English and Spanish for conduct by web (tablet, computer, or smartphone) or telephone with a trained interviewer. The questionnaire included items capturing demographics, existing health conditions, insurance coverage, employment, prior COVID-19 testing, exposure to COVID-19, symptoms experienced in the past three months, and attitudes and behaviors related to COVID-19 (e.g., social distancing, masking). We used Likert scales to assess both behaviors (never, rarely, some of the time, most of the time, all of the time) and attitudes (very important, important, little important, not important). Verbal consent was obtained from participants prior to completion of the questionnaire by a trained telephone interviewer. Online participants gave electronic consent after reading the consent material but prior to beginning the questionnaire.

After completing the questionnaire, participants scheduled COVID-19 PCR and antibody testing (via the Abbott Alinity platform, with high sensitivity and specificity) [27] to evaluate active and past infection. Written consent was obtained from all participants prior to COVID-19 testing. Given the large geographic area of the two counties (~1,800 square miles), the study team used four existing testing sites and established 11 new community-based testing sites across Dallas and Tarrant Counties, particularly in underserved neighborhoods, to provide convenient testing for study participants. We also deployed mobile phlebotomists in Dallas County for participants not wishing or able to visit a testing site.

Participants who completed the questionnaire but never scheduled an appointment or those who missed their scheduled appointment were called up to four times to reschedule. PCR and antibody test results were mailed within two weeks of test completion, and participants with active infection were called within 48 hours. Participants were provided $20 honoraria with the mailed test results. Testing spanned July 2020 to early March 2021.

### Community engagement

An internal advisory board, including specialists in infectious disease, epidemiology, laboratory medicine, and community-engaged research, provided input on the initial study design. In addition, a 32-member Community Advisory Board (CAB) representing government, business, non-profit, school, faith-based, and minority-serving civic organizations from both counties met monthly to provide feedback. The CAB advised on study design and recruitment materials, helped raise study awareness among minority and low-income communities through their personal and professional networks, and disseminated study findings.

### Data preparation and statistical analyses

Data were evaluated for quality prior to construction of the survey analysis weights and other analytic variables. We replaced missing responses for sex, age, race, ethnicity, education, income, and number of adults within household (each <6% missing) using one of two methods: logical assignment based on other information in the questionnaire and statistical imputation via a Hot-Deck method [28]. We created three sets of weights: one set of *household base weights* calculated as the inverse selection probability for the address, and two sets of weights adjusted to limit biases in population estimates—one set for the questionnaire responses and one set for the testing results. The *questionnaire weights* comprised the household base weight, a household nonresponse adjustment, an inverse within-household selection probability for the selected adult, and a calibration adjustment to align the person-level weights to population totals from the American Community Survey by sex, age category, race, ethnicity, and educational attainment within each county [25, 29]. The *COVID-19 testing weights* were derived by re-calibrating the questionnaire weights for those completing testing to the same population totals used previously to adjust for testing nonresponse. All nonresponse and calibration weight adjustments were calculated using the WTADJUST procedure in SUDAAN^®^ [30].

Unweighted analyses were conducted to describe sample characteristics. Weighted response rates were calculated for the questionnaire and COVID-19 testing components for context with other studies. Questionnaire response rates were calculated as the weighted percentage of completed questionnaires for all non-vacant study addresses using the household base weights. COVID-19 testing response rates were calculated as the weighted percentage of completed tests (PCR and antibody) for those with a completed questionnaire using the questionnaire weights.

We estimated the weighted prevalence of COVID-19 infection, defined as a positive PCR or antibody test, overall and by county, neighborhood-level SES, sex, race/ethnicity, age, essential worker status, and education. While we performed statistical comparisons of COVID-19 prevalence between subgroups of interest, survey data regarding attitudes and barriers were reported descriptively across subgroups without formal statistical comparisons. Log binomial regression models were used to examine the adjusted impact of sex and age on COVID-19 infection and to report adjusted prevalence ratios. All weighted analyses were conducted with SAS version 7.1 (Cary, North Carolina) survey procedures using the specified analysis weight, accounting for the stratified random sampling design.

## Results

### Participation

From 54,209 addresses randomly selected for the study, 2,969 adults completed the questionnaire (7.4% weighted response). Approximately 92% of the participants who completed the questionnaire and 89% who completed COVID-19 testing did so by the end of October 2020, just prior to the COVID-19 surge in the DFW area. Questionnaire response rates were slightly higher for Dallas versus Tarrant County (7.8% vs. 6.8% weighted). Among questionnaire respondents, 1,772 adults (53.9% weighted) completed COVID-19 testing, with a lower proportion for Dallas versus Tarrant County (52.5% vs. 55.6% weighted). The median interval between questionnaire and testing completion was 4.9 (IQR 2.8–8.7) days.

**Table 1** shows characteristics of participants completing the questionnaire and testing. The most common age group was 45–64 years, 38.2% were men, and 37.6% of participants resided in low SES CBGs. Most participants were NH White (49.4%), married (45.1%), had some type of health insurance (86.5%), resided in a household without children (75.6%), and held at least a 4-year college degree (47.8%). Additionally, most did not self-report as being an essential worker (71.1%). Approximately 59.7% of questionnaire respondents completed COVID-19 testing, with a higher proportion of participants with at least a 4-year college degree (69.8%), who were NH White (66.5%), and age 45–64 years (62.9%) completing testing. The lowest participation in testing completion was among participants with less than a high school education (37.8%) or a high-school level degree (44.9%) and those without health insurance (48.5%).

**Table 1:** Distribution of participants by select characteristics.

		Completed Questionnaire		Completed COVID-19 Tests		
		n	pct^a^	n	Percent among all tests by category	Percent among completed questionnaires by category
**Dallas and Tarrant counties (Total)**		2,969	100	1,772	100	59.7
**Characteristic (category)**						
**County**	**Dallas**	1,640	55.2	966	54.5	58.9
	**Tarrant**	1,329	44.8	806	45.5	60.6
**Neighborhood**	**Low SES**	1,116	37.6	609	34.4	54.6
	**Not Low SES**	1,853	62.4	1,163	65.6	62.8
**Sex**	**Male**	1,135	38.2	710	40.1	62.6
	**Female**	1,834	61.8	1,062	59.9	57.9
**Race and Ethnicity** ^**a**^	**Hispanic**	576	19.4	309	17.4	53.6
	**NH Black**	732	24.7	379	21.4	51.8
	**NH White**	1,467	49.4	976	55.1	66.5
	**NH Other**	194	6.5	108	6.1	55.7
**Age Group (Years)**	**18–24**	178	6.0	91	5.1	51.1
	**25–44**	925	31.2	520	29.3	56.2
	**45–64**	1,038	35.0	653	36.9	62.9
	**65–89**	828	27.9	508	28.7	61.4
**Marital Status**	**Married / Cohabit**	1,328	45.1	815	46.1	61.4
	**Single, never married**	796	27.0	465	26.3	58.4
	**Other**	819	27.8	488	27.6	59.6
**Health Insurance**	**Yes**	2,548	86.5	1,576	89.1	61.9
	**No**	396	13.4	192	10.9	48.5
**Children in Household**	**Yes**	714	24.4	371	21.1	52.0
	**No**	2,218	75.6	1,391	78.9	62.7
**Essential Worker** ^b^	**Yes**	809	28.9	480	27.8	59.3
	**No**	1,988	71.1	1,246	72.2	62.7
**Educational Attainment** ^c^	**< HS**	180	6.1	68	3.8	37.8
	**HS degree**	537	18.1	241	13.6	44.9
	**Some college**	833	28.1	473	26.7	56.8
	**College degree**	1,419	47.8	990	55.9	69.8

n = sample count; percent = unweighted percent

^a^ Non-Hispanic (NH) Other is comprised of racial groups other than White and Black including multiracial.

^b^ Essential workers (Yes) are defined as those who worked onsite in the past 30 days at the time of the survey most of the time and who came into frequent contact with the public. The ‘No” category includes those not working (e.g., student, retired).

^c^ < HS (high school) = less than high school/ high school no diploma; HS = high school, vocational, business or trade school; some college = some college no degree/associate degree; college degree = 4-year college/university degree/graduate school

### COVID-19 infection prevalence

**Table 2** shows the estimated prevalence of COVID-19 infection for the adult population in Dallas and Tarrant counties. Overall, 11.5% of adults had either active or prior COVID-19 infection, with a similar prevalence in both counties. In adjusted analyses (including age and sex), the prevalence for Hispanic and NH Black adults was 4.9 and 2.7 times higher (PR 4.86; 95%CI 4.82–4.91 and PR 2.69; 95%CI 2.66–2.71; respectively) compared to NH Whites. The prevalence of COVID-19 was also 1.5 times higher for those residing in low SES neighborhoods (adjusted PR 1.46; 95%CI 1.45–1.47). Infection prevalence among those aged 25–44 years and 45–64 years was 3.0 and 1.8 times higher (PR 3.0; 95%CI 2.99–3.07 and PR 1.8; 95%CI 1.78–1.83; respectively) than among those aged 18–24 years. Compared to those with a college education, COVID-19 infection was 2.5, 7.1 and 2.3 times higher for adults with a degree less than high school, high school, and some college, respectively (PR 2.54; 95%CI 2.52–2.57; PR 7.08; 95%CI 7.02–7.15; and PR 2.27; 95%CI 2.25–2.30; respectively). Finally, infection was 1.4 times higher for self-reported essential workers (PR 1.43; 95%CI 1.42–1.44) compared to others.

**Table 2:** Estimated prevalence of COVID-19 infection by select characteristics.

Characteristics		Infection Prevalence Rate^a^		Adjusted Prevalence Ratio (95%CI)^b^
		Pct^c^	95% CI^d^	
**Dallas and Tarrant counties (Total)**		11.5	(7.89, 15.11)	
**County**	**Dallas**	12.1	(6.73, 17.46)	Reference
	**Tarrant**	10.8	(6.16, 15.34)	0.90 (95%CI 0.90–0.91)
**Neighborhood**	**Not Low SES**	9.7	(5.49, 13.82)	Reference
	**Low SES**	16.1	(9.01, 23.27)	1.46 (95%CI 1.45–1.47)
**Sex**	**Female**	13.0	(7.88, 18.06)	Reference
	**Male**	9.9	(4.85, 15.03)	0.87 (95%CI 0.87–0.88)
**Race and Ethnicity** ^e^	**NH White**	4.6	(2.4, 6.88)	Reference
	**Hispanic**	23.0	(13.43, 32.57)	4.86 (95%CI 4.82–4.91)
	**NH Black**	11.3	(5.06, 17.52)	2.69 (95%CI 2.66–2.72)
**Age Group (Years)**	**18–24**	5.2	(0, 11.65)	Reference
	**25–44**	16.6	(9.21, 23.89)	3.03 (95%CI 2.99–3.07)
	**45–64**	9.8	(5.19, 14.44)	1.81 (95%CI 1.78–1.83)
	**65–89**	6.4	(2.15, 10.66)	1.20 (95%CI 1.18–1.22)
**Essential Worker** ^f^	**No**	10.0	(6.05, 13.9)	Reference
	**Yes**	15.1	(7.36, 22.77)	1.43 (95%CI 1.42–1.44)
**Education** ^g^	**< HS**	33.1	(16.2, 50.06)	2.54 (95%CI 2.52–2.57)
	**HS degree**	10.2	(3.73, 16.73)	7.08 (95%CI 7.02–7.15)
	**Some college**	9.3	(4.92, 13.72)	2.27 (95%CI 2.25–2.30)
	**College degree**	4.7	(2.87, 6.62)	Reference

n = sample count; NH = non-Hispanic

^a^ Positive PCR or antibody test. Inconclusive results classified as a negative result.

^b^ Adjusted for age and sex.

^c^ Survey weighted percent of those who were infected.

^d^ Survey weighted 95% confidence interval.

^e^ The unstable ’NH Other’ estimate, which includes NH Other and NH Multiracial adults, was suppressed.

^f^ Essential workers (Yes) are defined as those who worked onsite in the past 30 days at the time of the survey most of the time and who came into frequent contact with the public. The ‘No” category includes those not working (e.g., student, retired).

^g^ < HS = less than high school/ high school no diploma; HS = high school, vocational, business or trade school; some college = some college no degree/associate degree; college degree = 4-year college/university degree/graduate school

### Attitudes about recommended COVID-19 health behaviors

**Fig 1:**
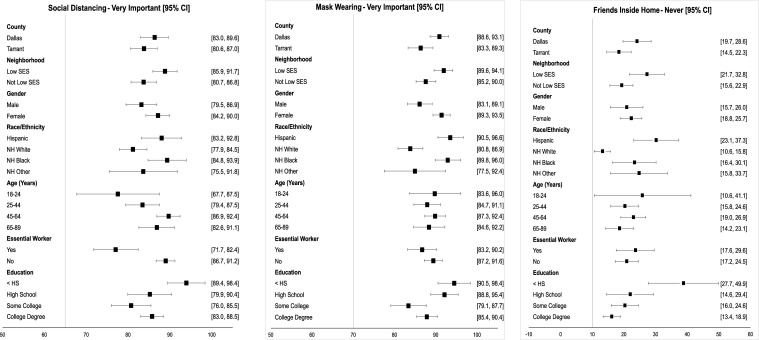
Estimated prevalence by select beliefs that lower risk of COVID-19 infection. SES = Socioeconomic status. Non-Hispanic (NH) Other is comprised of racial groups other than White and Black including multiracial. Essential workers (Yes) are defined as those who worked onsite in the past 30 days at the time of the survey most of the time and who came into frequent contact with the public. The ‘No” category includes those not working (e.g., student, retired). Education: < HS = less than high school/ high school no diploma; HS = high school, vocational, business or trade school; some college = some college no degree/associate degree; college degree = 4-year college/university degree/graduate school.

Overall, most respondents reported that recommended health behaviors were very important to decrease the risk of infection: social distancing (85.2%) and wearing masks (89.0%)—see **Fig 1, Panels A and B**, respectively. However, belief in the importance of social distancing varied by participant characteristics.

A lower proportion of essential workers (77.1%) and young (age 18–24) adults (77.6%) felt that social distance was very important compared to their counterparts. The belief that mask wearing was very important had less variation but was highest for adults with some college education (94.5%) and lowest for those with less than a high school degree (86.7%). NH White and NH Other adults were slightly less likely to consider social distancing and mask wearing very important compared to Hispanic and NH Black adults; the same pattern was found for persons in low SES CBGs vs. other and for males vs. females.

The proportion never hosting friends inside the home (**Fig 1, Panel C**) was lowest among the three attitudes evaluated: 22.6% overall, ranging from 13.2% (non-Hispanic Whites) to 25.8% (age 18–24 years).

### Adoption of CDC recommendations to prevent COVID-19 spread

**Fig 2:**
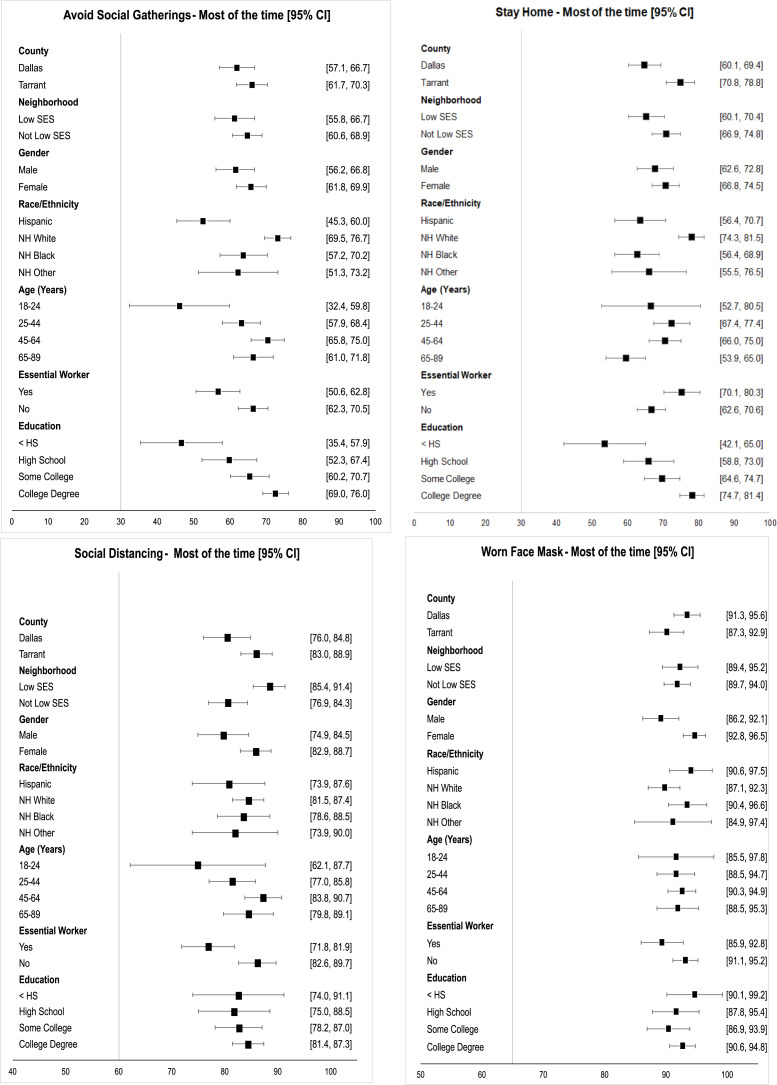
Estimated prevalence by select behaviors that lower risk of COVID-19 infection. SES = Socioeconomic status. Non-Hispanic (NH) Other is comprised of racial groups other than White and Black including multiracial. Essential workers (Yes) are defined as those who worked onsite in the past 30 days at the time of the survey most of the time and who came into frequent contact with the public. The ‘No” category includes those not working (e.g., student, retired). Education: < HS = less than high school/ high school no diploma; HS = high school, vocational, business or trade school; some college = some college no degree/associate degree; college degree = 4-year college/university degree/graduate school.

**Fig 2** describe adoption of CDC recommendations to prevent infection: Limiting social gatherings, staying home unless necessary, keeping at least a 6-foot “social distance” away from those not residing in their household, and wearing a mask outside the home. In general, a higher proportion of women practiced recommended behaviors than men. Except for mask wearing, a lower proportion of young adults practiced recommended behaviors.

Nearly two-thirds (62.3%) reported avoiding social gatherings most of the time. A lower proportion of younger adults (age 18–24 years, 46.1%), participants without a high school degree (46.6%), and Hispanics (52.6%) reported avoiding social gatherings compared to those aged 45–64 years (70.4%), with at least a 4-year college degree (72.5%), and NH White (73.1%).

Similarly, 68.1% reported staying at home most of the time, with a higher proportion in Tarrant (74.8%) compared with Dallas (64.7%) County. The lowest percentage was for adults with less than a high school degree (53.5%) and older adults (59.5%). The proportion was also lower for NH Black (62.6%) and Hispanic (63.5%) adults compared with NH White adults (77.9%).

Overall, a high proportion of participants reported social distancing most or all of the time (82.7%). These values ranged from a low of 74.9% for young adults to 88.4% for those residing in low SES neighborhoods. Estimates for essential workers (76.8%) were lower than other adults in the population (84.4%).

The highest adoption of CDC guidelines was reported for wearing a face mask most of the time when not at home (92%). Differences across SES, age, and education were minimal. However, adoption was highest for women (94.7%), and estimates were below 90% for men, essential workers, and NH Whites.

## Discussion

In this study, we found that 11.5% of the adult population in Dallas and Tarrant counties had active or prior COVID-19 infection early in the pandemic–prior to the surge of infections that occurred in North Texas in winter 2020. Nearly 90% believed wearing face masks was very important and reported doing so most of the time. However, a lower proportion believed that social distancing was very important and many reported engaging in social gatherings. Public health communications should continue to emphasize the importance and efficacy [31] of social distancing and minimizing large gatherings to mitigate COVID-19 risk, especially given the current delta variant surge.

Similar to other studies, we observed sociodemographic disparities in COVID-19 infection, with higher prevalence among younger adults [7, 17]; Hispanics [11, 19, 32] and NH Blacks [4, 9, 19]; and those residing in low-income neighborhoods [32]. We also found disparities in adopting CDC recommendations across various sociodemographic groups. For example, lower adherence to public-health guidelines among young adults highlights the importance of interventions targeted to this age group. Moreover, we estimated attitudinal and behavioral differences such as NH Whites being less likely to believe in the importance of mask wearing whereas racial and ethnic minorities were more likely to attend large social gatherings.

Other seroprevalence studies conducted prior to summer/fall of 2020 and during shelter-in-place orders [9–11, 16, 33] found lower COVID-19 prevalence compared to our study. The exceptions are studies conducted in hotspots, like New York City and Chelsea, Massachusetts [19, 20], as well as ski resort communities [17]. Studies conducted during the summer/fall of 2020 estimated a similar prevalence [5–7, 18] and consistent patterns of higher infection among racial and ethnic minority [6, 7] and low-income participants.

Study results should be interpreted considering its limitations. First, data were collected from most participants between July and October 2020, before the major surge in COVID-19 infection in Dallas and Tarrant counties, and related behaviors and attitudes continued to evolve over time. Second, nonresponse bias (i.e., if adults more compliant with CDC recommendations were more likely to respond) in the study estimates may not have been fully addressed through weighting adjustment. Third, estimates for behaviors and attitudes may have been influenced by social desirability bias where respondents provide answers that align with CDC recommendations and not necessarily their actual attitudes or practiced behaviors. Finally, antibody waning may lead to an underestimate in the cumulative prevalence of infection [34].

These limitations are contrasted with the study’s many strengths: collection of COVID-19 attitudes and behaviors paired with testing results in a racial, ethnically, and socioeconomically diverse cohort on a random sample of adults. As world-wide disease outbreaks continue to increase [35–38], the need for effective protocols to monitor real-time population-based estimates is vital, and our study also serves this purpose. The weighted response rate (7.4%) to our mailed study invitation was less than expected—though comparable to other COVID-19 prevalence studies [6–8] and higher than the U.S. Census Bureau’s *Household Pulse Survey* (5.5%) [39]—limiting power to assess differences among subgroups. In-person recruitment may have increased participation (e.g., 23.7% response rate for Biggs [9]), but resources were not available for this intensive effort.

Others collected timely data on attitudes and behaviors from existing online survey panels [40, 41], at the exclusion of biospecimens to confirm infection. Consequently, we conducted a second protocol using nonprobability opt-in strategies [4, 17, 19, 42] for rapid recruitment and timely data collection to provide prevalence estimates, paired with data describing behaviors and attitudes, that address public health needs [43]. In the interim, our findings suggest differences in COVID-19 attitudes and behaviors likely contribute to sociodemographic disparities in prevalence. These data highlight the importance of continued public health measures, including social distancing and minimizing large gatherings, to mitigate COVID-19 morbidity.

## Supporting information

S1 Data (XLSX)

S1 File (DOCX)
