# Supplementary material for: Population-based correlates of COVID-19 infection: An analysis from the DFW COVID-19 prevalence study
Source: PLoS One. 2022 Dec 1;17(12):e0278335. doi: 10.1371/journal.pone.0278335 (PMC9714738; doi:10.1371/journal.pone.0278335)
Supplement: S1 File — (DOCX) [file pone.0278335.s002.docx]

**DFW COVID-19 PREVALENCE STUDY QUESTIONNAIRE**

The University of Texas Southwestern Medical Center (UT Southwestern) and Texas Health Resources, in partnership with RTI International, are conducting The DFW COVID-19 Prevalence Study to find out how many people currently have or have been exposed to COVID-19 in the Dallas-Fort Worth area.

You will first answer some questions taking about 15 minutes on your demographics and behaviors, possible symptoms, and past tests related to the COVID-19 pandemic. Then, you will schedule an appointment for your free COVID-19 testing on the UT Southwestern registration site.

You will receive $20 after the COVID-19 test is completed and results of the tests after they are analyzed.

2/CONFIRM_ADDR Is this your address? [FILL: ADDRESS].

- 1. Yes
  2. No

3. What is your first and last name?

3A/FIRST_NAME. First name ___________________ [ALLOW 40]

3B/LAST_NAME. Last name ___________________ [ALLOW 60]

3C/FON_FR_SRV Please provide a phone number with area code in case we need to contact you about your responses.

Phone number: _______________ [10 DIGITS]

4/DOB What is your date of birth?

[ _ _ /_ _ / _ _ _ _] *(MM/DD/YYYY)*

5/GENDER. What is your sex?

- 1. Male
  2. Female

6/HISPANIC. Are you of Hispanic, Latino, or Spanish origin?

- 1. No, not of Hispanic, Latino, or Spanish origin
  2. Yes

7/RACE What is your race? *Please select all that apply.*

- 1. White
  2. Black or African American
  3. American Indian or Alaska Native
  4. Asian
  5. Native Hawaiian or Pacific Islander
  6. Other, please specify: ________________ [ALLOW 40]

8/LANGUAGE What language do you most commonly speak at home?

- 1. English
  2. Spanish
  3. Other, please specify: ________________ [ALLOW 40]

9/MARITAL_STAT. What is your current marital status?

1. Married
2. Living with partner
3. Widowed
4. Divorced
5. Separated
6. Single, never married

10/TOTAL_ADULTS Including yourself, how many adults aged 18 or older live in your home?

[ALLOW 1-20]

10A/TOTAL_MALE [IF Q5=1 (MALE), Including yourself, how | ELSE How] many adults 18 years of age or older living in your home are male?

[ALLOW 0-20]

1 Change my answer for the number of males living in my home.

2 Change my answer for gender.

3 Prefer not to answer.

10B/TOTAL_65 Including yourself, how | ELSE How] many adults living in your home are 65 years of age or older?

[ALLOW 0-20]

11/TOTAL_CHILD How many children under the age of 18 currently live in your home? Consider all children under the age of 18 who live with you, even temporarily.

[ALLOW 0-20]

AGE_1_CHILD How old is this child?

1 AGE 4 OR LESS

2 AGE 5 THROUGH 12

3 AGE 13 THROUGH 17

4 Prefer not to answer

11A. How many of the [Q11 RESPONSE] children living in your home are in the following age ranges?

1/CHLD_4_LESS AGES 4 YEARS OR LESSAges 4 years or less: [ ]

2/CHLD_5_12 AGES 5 THROUGH 12: [ ]

3/CHLD_13_17 AGES 13 THROUGH 17: [ ]

11B/CHILDCARE Since March 2020, have you used any services to care for your children such as daycare, nanny or babysitter?

1. Yes

2. No

11C/CHLD_ACTIV Since March 2020, have any of the children in your home participated in group activities, such as sports, camps, or Sunday school, with 5 or more other children?

1. Yes

2. No

12/COVID_TESTED Have you been tested for COVID-19 by a doctor or health care professional?

- 1. Yes – one time
  2. Yes – more than one time
  3. No

12A/TEST_MONTH What month in 2020 were you tested? If you have been tested more than once, please enter the month of your **most recent test**.

1 March

2 April

3 May

4 June

5 July

6 August

7 September

8 October

9 November

10 December

12B/TEST_RESULTS What were the results of your most recent COVID-19 test?

1 I had COVID-19

2 I did NOT have COVID-19

3 The test could not determine if I had COVID-19

4 I am waiting for the results.

12C/PRIOR_RESULT Did any of the prior COVID-19 tests produce a positive or inconclusive result?

1 Yes

2 No

12D/PR_TRY_TEST Have you previously tried to get tested for COVID-19?

1 Yes

2 No

13/SYMPTOMS_2W **In the past 2 weeks**, have you had any of the following symptoms? *Please select all that apply.*

1 Fever

2 Chills

3 Shortness of breath or trouble breathing

4 Cough

5 Sore throat

6 New loss of smell or taste

7 Muscle aches or body aches

8 Vomiting

9 Diarrhea

10 Fatigue

11 Headache

12 No symptoms

14/SYMPTOMS_3M **Excluding the** **past 2 weeks,** have you had any of the following symptoms in the **past 3 months**? For reference, this would be between [FILL DATE –90 DAYS] and [FILL DATE -14 DAYS]. *Please select all that apply.*

1 Fever

2 Chills

3 Shortness of breath or trouble breathing

4 Cough

5 Sore throat

6 New loss of smell or taste

7 Muscle aches or body aches

8 Vomiting

9 Diarrhea

10 Fatigue

11 Headache

12 No symptoms

15/CONTACT_POS The COVID-19 pandemic and shelter-in-place policies started in Dallas and Fort Worth in March 2020. **Since March 2020,** have you been in close contact, within 6 feet for more than 15 minutes, with a person who has tested positive for COVID-19?

1 Yes – one person

2 Yes – more than one person

3 No

4 Don’t know

16.CON_POS_RES What is your relationship to that person/ these people? *Please select all that apply.*

1 Household member

2 Co-worker

3 Neighbor

4 Friend

5 Other, please specify: ______________ [ALLOW 60]

17/CONTACT_SYMP **Since March 2020,** have you been in close contact, within 6 feet for more than 15 minutes, with a person who has had COVID-like symptoms? For example, fever, cough, shortness of breath, sudden loss of taste or smell.

1 Yes – one person

2 Yes – more than one person

3 No

4 Don’t know

18./CON_SYMP_REL What is your relationship to [Q17=1 that person / Q17=2 these people]? *Please select all that apply.*

1 Household member

2 Co-worker

3 Neighbor

4 Friend

5 Other, please specify: ______________ [ALLOW 60]

19./TRAVEL **Since March 2020,** have you traveled outside of the Dallas-Fort Worth metroplex for an overnight visit?

1 Yes

2 No

20A./TRAVEL_DEST Where did you travel? *Please select all that apply.*

1 To other cities within Texas

2 To other states within the continental U.S.

3 To another country

20B./TRAVEL_MODE When you travelled, what mode of transportation did you use? *Please select all that apply.*

1 Personal car

2 Public transportation (e.g. commercial airline, train, bus

3 Other, please specify: __________________ [ALLOW 80]

20C./TRAVEL_MONTH During your most recent trip, what month did you travel?

1 March

2 April

3 May

4 June

5 July

6 August

7 September

8 October

9 November

10 December

21. **Since March 2020,** how often did you do the following behaviors?

|  | | | | |  |
| --- | --- | --- | --- | --- | --- |
|  | Never | Rarely | Some of the time | Most of the time | Not Applicable |
| - - - 1. BEHAVIORS_1 Staying at home except for going to work, getting food, going out to exercise, or getting medical care |  |  |  |  |  |
| 1. BEHAVIORS_2Going to work even if you felt like you might be getting sick |  |  |  |  |  |
| 1. BEHAVIORS_3Staying 6 feet away from people when you leave your home |  |  |  |  |  |
| 1. BEHAVIORS_4Wearing a face mask when not at home |  |  |  |  |  |
| 1. BEHAVIORS_5Washing your hands with soap and water or using alcohol-based hand cleaners after returning home from anywhere |  |  |  |  |  |
| 1. BEHAVIORS_6Outside of work, avoiding gatherings with more than 5 people who do not live in the same house as you |  |  |  |  |  |
| 1. BEHAVIORS_7Avoiding public transportation (e.g. DART, buses, Uber, flights) |  |  |  |  |  |
| 1. BEHAVIORS_8Having relatives, friends, or neighbors come into your home |  |  |  |  |  |
| 1. BEHAVIORS_9Having workers (e.g. cable company, pest control, repair technician) come into your home |  |  |  |  |  |

21/SOCIAL_DIST How **important** do you think social distancing is during COVID-19? Social (or physical) distancing means keeping space between yourself and other people outside your home.

1 Very important

2 Somewhat important

3 A little important

4 Not important

5 Don’t know/not sure

22/MASK_NHOME. How **important** do you think wearing a face mask is when outside your home and social distancing may be difficult?

1 Very important

2 Somewhat important

3 A little important

4 Not important

5 Don’t know/not sure

23/CONDITIONS Have you ever been told by a doctor or other healthcare professional that you had any of the following? *Please select all that apply.*

1 Heart disease 10 High blood pressure

2 Lung disease 11 Diabetes

3 Ulcer or stomach disease 12 Kidney disease

4 Liver disease 13 Anemia or other blood disease

5 Cancer 14 Rheumatoid arthritis

6 HIV 15 Neurologic disease

7. Stroke 16 Obesity

8 Currently pregnant 17 Immunocompromised, please specify ______ [ALLOW 40]

9 Auto-immune disease 18 Other, please specify ______ [ALLOW 40]

19 None: I have never been told by a doctor or other healthcare professional that I have a medical condition

24./HEALTH_COV Do you have health insurance or health coverage?

1. Yes
2. No

24A/TYPE_COVERAG What type of health insurance/coverage do you have? *Please select all that apply.*

1. Insurance plan paid for by current or former employer or union

2. Insurance plan that I, or family member, purchased directly from an insurance company (e.g., Blue Cross Blue Shield, Aetna)

3. Insurance through the state Health Insurance Exchange (HIE), called the Texas State HIE Plan. This is also known as “Obamacare”

4. Medicare, for people 65 years and older or people with certain disabilities.

5. Medicaid, for low-income children and their families, pregnant women, and disabled or elderly people

6. CHAMPUS/CHAMP-VA, TRICARE, VA or other military health care

7. Parkland Financial Assistance (PFA) or JPS Connect

8. Indian Health Service, Tribal Health Program, or Urban Indian Clinic

9. Other health insurance or health coverage, please specify: [ALLOW 40]

10. Do not know

25./HH_INCOME What was your total household income before taxes and deductions from all sources in **2019**?

1 $0 – $19,999

2 $20,000 - $34,999

3 $35,000 - $49,999

4 $50,000 - $64,999

5 $65,000 - $79,999

6 $80,000 - $99,999

7 $100,000 or greater

26./EDUCATION What is the **highest** grade or level of school you have **completed**?

1 Less than high school

2 Some high school but did not get diploma

3 High school degree or equivalent (e.g. GED)

4 Vocational, business, or trade school

5 Some college but no degree

6 Associate degree (junior or community college)

7 4-year college or university degree

8 Graduate or professional school (MA, MS, MBA, MD, PhD, etc.)

27/EMPLOYMENT. Are you currently…

1 Employed for wages

2 Self-employed

3 Unemployed

4 Temporarily unemployed/ furloughed

5 A homemaker

6 A student

7 Retired

8 Unable to work

27A./EMPLOY30D During the past 30 days, that is since [FILL DATE –30 DAYS], were you working at a job or business?

1 Yes

2 No

27B/NWORK_BUS During the past 30 days, that is since [FILL DATE –30 DAYS], did you have a job or business but were not working?

1 Yes

2 No

27C./PART_FULL Are you currently/Were you employed full-time or part-time?

1 Full time

2 Part time

27D./WORK_REMOTE Do you currently/Did you work on-site at the business or remotely from home?

1 Work on-site at the business

2 Remote from home

3 Combination of on-site and remote

4 Other, please specify: ________________ [ALLOW 40]

27E/WORK_CONTACT While at work during the past 30 days, that is since [FILL DATE –30 DAYS], how often do/did you come into close contact, within 6 feet for more than 15 minutes, with other people?

1 All of the time

2 Most of the time

3 Some of the time

4 Rarely

5 Never

27F./OCCUPATION What is your occupation?

____________________ [ALLOW 200]

27G/OCC_GROUP. Which of the following groups best describes your occupation?

1 Management, business and financial operations

2 Computer and mathematical

3 Architecture and engineering

4 Life, physical, and social sciences

5 Community and social services

6 Legal

7 Education and library (including daycare or childcare services)

8 Arts, design, entertainment, sports, media

9 Healthcare practitioners, staff, and technical support

10 Protective services (e.g. police, fire, EMT)

11 Food preparation, serving, and delivery

12 Building and grounds cleaning and maintenance

13 Personal care and service (e.g. hair salon, nail, massage)

14 Sales and related occupations

15 Office and administrative support occupations

16 Construction and extraction

17 Farming, fishing, forestry

18 Installation, maintenance, repair

19 Production

20 Transportation, material moving

21 Warehousing and storage (e.g., Amazon)

22 Military-specific occupations

23 Other, please specify: ________________ [ALLOW 60]
